# Pathways of healthcare utilisation in patients with suspected adolescent idiopathic scoliosis: a cross-sectional study

**DOI:** 10.1186/s12913-015-1152-1

**Published:** 2015-11-07

**Authors:** Marie Beauséjour, Lise Goulet, Debbie Ehrmann Feldman, Roxane Borgès Da Silva, Raynald Pineault, Michel Rossignol, Marjolaine Roy-Beaudry, Hubert Labelle, Scoliosis Referral Project members

**Affiliations:** Research Centre, Sainte-Justine University Hospital Centre, 3175 Côte Sainte-Catherine, Montreal, Quebec H3T 1C5 Canada; University of Montreal Public Health Research Institute (IRSPUM), P.O. Box 6128, Centre-Ville Station, Montreal, Quebec Canada; Department of Surgery, Faculty of Medicine, University of Montreal, P.O. Box 6128, Centre-Ville Station, Montreal, Quebec Canada; Agence de la santé et des services sociaux de Montréal, 1301 Sherbrooke St. E., Montreal, Quebec H2L 1M3 Canada; Department of Epidemiology Biostatistics and Occupational Health, McGill University, Montreal, Quebec H3A 0G4 Canada

**Keywords:** Healthcare pathways, Appropriateness of care, Referral, Healthcare utilisation, Adolescent idiopathic scoliosis

## Abstract

**Background:**

School screening programs for adolescent idiopathic scoliosis (AIS) have been discontinued in Canada and elsewhere because they were not considered cost-effective. In communities lacking such programs, we expect a significant variety of healthcare pathways and timeframes for patient referrals to orthopaedics.

The objectives of this study were: 1) to characterise the healthcare pathways of young children with suspected AIS in a population without school screening; and 2) to investigate the relationships between these healthcare pathways and the appropriateness of referrals to specialised orthopaedic clinics.

**Methods:**

This study concerned all children, ages 10 to 18, referred for an initial visit for suspected AIS to any of the five out-patient paediatric orthopaedic clinics of south-western Quebec (Canada). For the 831 participants, referrals to orthopaedics were characterised as appropriate, late, or inappropriate, based on known risk factors for AIS progression and on treatment indications. Parents documented the circumstances of healthcare use prior to the orthopaedic consultation. Relevant predisposing, enabling, and need variables derived from Andersen’s Behavioral Model of Health Services Use were also documented. Healthcare pathways were characterised by developing a taxonomy using multiple correspondence analysis prior to hierarchical classification. Associations between the healthcare pathways and appropriateness of referral were assessed using multinomial regression analyses.

**Results:**

We constructed a taxonomy of five distinct healthcare pathways: 1) Lay/regular source of care interrelation, 2) Other professionals, 3) Lay/consultation discontinuity, 4) Other medical doctor, and 5) Regular source of care continuity. Laypersons played an important role in AIS suspicion (53 % of cases), but did not prevent late referrals. Continuity of care, as opposed to numerous uncoordinated consultations, was an effective strategy to prevent late referrals (OR = 0.32 [0.17–0.59]), but was related to increased probability of inappropriate referrals.

**Conclusions:**

We identified two cardinal characteristics that distinguished the healthcare pathways and related significantly to appropriateness of referral status, namely the role of laypersons and the involvement of the regular source of care. This suggests directions for intervention such as advocating for access to a regular source of care, increasing awareness of the disease to medical practitioners’ and improving their knowledge of AIS detection and referral criteria.

## Background

Screening programs for adolescent idiopathic scoliosis (AIS) were instituted in many school settings in the 1970s. They consisted of back examinations of young adolescents, usually performed by a specially trained nurse searching for asymmetries in standing and forward bending positions (Adams Forward Bending Test) [1–5]. These programs were progressively discontinued in Canada and in many other countries because they were not considered a cost-effective preventive measure [6–9]. Considering the state of knowledge at that time, the ability of the detection procedure to identify the target condition and the ability of the treatment intervention to achieve a favorable outcome were questioned by the national task forces. Indeed, screening programs resulted in the referral of large numbers of patients without significant curves. In addition, screening programs did not change patients’ management since early brace treatment had not yet been proven to be effective [6, 7]. The latest Canadian position is that insufficient evidence exists to make a recommendation for or against the screening programs [10]. Although programs were discontinued, it has nevertheless been suggested, both by public health specialists and by physicians, that back inspection should remain part of family physicians’ regular examination of pre-adolescents [2, 7, 11].

In the context of school scoliosis screening programs healthcare resource use was predictable, including referral patterns of suspected AIS cases. Indeed, the programs ensured that the vast majority of patients were accessing diagnostic facilities and specialised orthopaedic care early [2, 3], and almost all known scoliosis cases came from these screening facilities [12]. In communities lacking such programs, we can now expect a wide variety of healthcare pathways and of timing of patients’ referral to orthopaedics [13].

In the absence of school screening programs, no data have been collected on how suspected AIS cases are managed in the primary care setting, nor on the circumstances under which they are referred to an orthopaedist. Descriptive data do exist in Canada, England and Norway, on the characteristics of patients referred for suspected AIS in orthopaedic clinics [13–17]. These studies on the case-mix have shown significant rates of both over-referral and late referral [13–17], suggesting that current referral mechanisms are suboptimal. Factors associated with timing of referral in AIS include gender, age, and type of scoliosis curves [13], as well as family history of scoliosis and the originator of the detection [14, 15, 18, 19].

Delay in referral may imply that patients are referred with large curves precluding conservative treatment by orthopaedic brace, or significantly decreasing the likelihood of treatment success [20]. This situation is of particular concern considering the recent publication of the results of the BrAIST study demonstrating the effectiveness of early management by bracing [21]. Such findings emphasise the need to adjust referral mechanisms to maximise the benefits of this treatment modality. On the other hand, inappropriate referrals lead to unnecessary use of specialised healthcare resources, resulting in longer waiting times and reducing orthopaedic consultants’ availability to concentrate on complex cases. Current referral mechanisms raise questions regarding the medical benefits of healthcare intervention, equity, the best choice of intervention setting, and cost-effectiveness—all dimensions related to appropriateness of healthcare utilisation [22].

In this study, our overall aims were: 1) to characterise the healthcare pathways of young children with suspected AIS in a population without school screening or any specific intervention program for early detection; and 2) to investigate the relationships between these healthcare pathways and the appropriateness of referral to specialised orthopaedic clinics. A healthcare pathway is defined as the patient’s journey through the primary healthcare system from scoliosis suspicion or detection to the referral consultation, including all encounters with medical doctors, allied health professionals or alternative caregivers, for investigation, provisional diagnosis, management, and referral.

### Conceptual framework

**Fig. 1:**
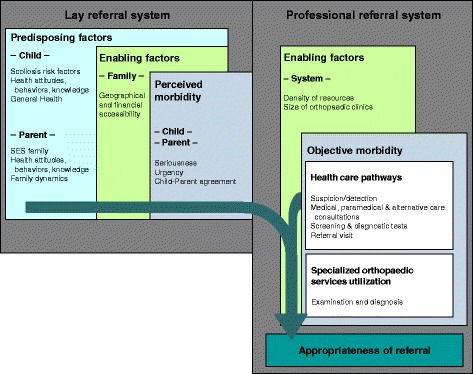
Conceptual framework for the study of the determinants of the appropriateness of referral. SES: Socio-economic status

The conceptual framework for studying the relationships between user (child and family) and system characteristics, primary healthcare utilisation (healthcare pathways), and outcomes (appropriateness of referral to a specialised orthopaedic clinic) is derived from Andersen’s Behavioral Model of Health Services Use [23–25] and is presented in Fig. 1.

This well validated and largely used model was chosen because it proposes an explanatory process and a logic organisation of the determinants of healthcare use. Considering the two objectives of the study, it is an appropriate choice of framework. It allows taking into account all relevant factors in order to characterise the pathways (objective 1). Indeed, it views access to services as a result of decisions made by an individual (and family), which are constrained by the social environment, and the availability of healthcare services [26]. It allows the investigation of the relationships between prior use and current patterns of health care services utilisation (objective 2).

In this model, predisposing, enabling, and need factors contribute to the individual’s health utilisation behaviour. Predisposing factors exist before the perception of morbidity and include demographic and social structural variables [27]. Enabling factors are individual or situational characteristics that facilitate or impede the utilisation of health services, such as individual and familial resources, as well as availability and accessibility of health services in a community [23–27]. Need factors are divided into two conceptual categories within our framework: those pertaining to the signs and symptoms of a health problem are labelled as perceived morbidity, and those referring to professional evaluation of the disease and primary healthcare utilisation, as objective morbidity. The latter is studied in relation to outcome, adjusting for predisposing, enabling, and perceived morbidity factors, as these may change the original relationships.

## Methods

### Study design and population

We conducted a cross-sectional study with all children aged between 10 and 18 years, and their accompanying parent (guardian), referred for an initial visit for suspected scoliosis in all five out-patient paediatric orthopaedic clinics of south-western Quebec (Canada) between February 2006 and August 2007. The study concerns five out of the six paediatric orthopaedic clinics in the province of Québec (a Canadian province of 7.5 million inhabitants). Therefore, it represents approximately 70 % of all patients seen in paediatric orthopaedic clinics in Québec. The population served by these clinics is ethnically and socioeconomically heterogeneous and comes from diverse regions (metropolitan, urban and rural areas) of the province. Quebec is a province without school screening or any specific intervention program for early scoliosis detection. Quebec’s health system is universal, with consultation in primary and specialised care fully covered by the government. A request by a referring physician is usually required to access specialised care. However, self-referral still occasionally occurred at participating clinics, which did not systematically require a referral letter when scheduling an appointment.

### Data collection

The research protocol and the questionnaires satisfied the ethical requirements of the institutional review boards of all the participating hospitals and institutions (Sainte-Justine University Health Centre, Shriners Hospital for Children, McGill University Health Centre, McGill University Faculty of Medicine, University of Sherbrooke Health Centre, and Children Hospital of Eastern Ontario). Newly referred patients at the spine clinics were identified from scheduled appointment lists. Children and accompanying parents were informed of the details of the research protocol, including voluntary participation and the protocol’s risks and benefits, by a research nurse or assistant. They were given adequate time to ask questions and review the information. If they agreed to participate, they were invited to sign a consent (assent) form and were given the questionnaires in a room adjacent to the clinic. Questionnaires were consistently administered before radiographic and medical examination to blind the interviewer to the outcome and prevent changes in the respondents’ disease perception.

Children were instructed to complete a self-administered questionnaire on their health perception, back signs, symptoms, and life habits. Their accompanying parent was first asked to participate in a face-to-face interview with a trained research assistant to document the detailed circumstances of healthcare use for their child’s back problem prior to the orthopaedic consultation. In addition, the parent completed a self-administered questionnaire focusing on family demographic and socio-economic characteristics as well as on the parent’s health-related attitudes, behaviours, and knowledge. Mean duration for questionnaire completion was 30 min. In some cases where time constraints impeded completion of questionnaires, permission was requested to access the respondent’s clinical record, to assess the possibility of a selection bias. All questionnaires were available in French and English, according to the participants’ preferences. All data were stored in a dedicated Access database (Microsoft Corp.), using unique numerical identifiers for confidentiality.

### Study variables

The main outcome in this study, appropriateness of referral, which determines orthopaedics utilisation, was assessed by comparing the child’s back condition at the time of referral with defined criteria of appropriateness [13] based on expert opinions, known risk factors for scoliosis progression [28–31], treatment indications [32–35], and published guidelines for the management of scoliosis patients [36–39]. Generally speaking, patients who should receive particular attention from an orthopaedist are those presenting a clinically significant scoliosis (as measured by the Cobb angle) and a residual growth potential (Risser sign) [40–45]. Therefore, the outcome was defined as a nominal variable consisting of three mutually exclusive categories: appropriate referral, inappropriate referral, and late referral. Appropriate referrals were those respecting the Scoliosis Research Society’s diagnostic criteria: lateral deviation of the spine above 10 degrees, without inherited disorders of connective tissue, neurological disorders, or other musculoskeletal disorders [36, 37], and where the referral was not late. Late referrals occur when skeletal maturity and curve magnitude at the initial visit in orthopaedics are beyond the indications for brace treatment (suggesting the need for surgical management) or are less likely to respond to treatment [29, 32, 34, 37, 46]. Therefore, patients presenting with a Cobb angle greater than 40°, regardless of skeletal maturity, and immature patients (Risser sign of 0, 1, 2, or 3) with a Cobb angle greater than 30° were all considered late referrals. Inappropriate referrals were those patients with curve magnitude below the diagnostic criteria, i.e., 10 degrees or less.

The main independent variables that characterise healthcare pathways are defined by five steps, which are nominal variables documenting primary healthcare utilisation prior to the orthopaedic consultation.

#### Suspicion

The circumstances under which scoliosis was first suspected or detected, i.e., whether it was by a layperson (a parent, a family member, a school teacher, a person in charge of an extra-curricular activity, a friend, or the child him/herself), or a health professional (the child’s regular source of care, another medical doctor, or another non-MD professional).

#### First medical consultation

In cases where the scoliosis suspicion was not raised by a medical doctor, the first medical consultation (by the regular source of care, another medical doctor, or never) was documented. This variable was intended to identify the physician who may have first provided a provisional diagnosis of scoliosis and to document the earliest opportunity along the healthcare pathway where a referral to orthopaedics could have been made.

#### Other consultations

This variable represents any other consultations with a healthcare professional reported by the parent for the back problem, that took place between the date of suspicion and the date of referral. This includes visits to the regular source of care and to any medical doctors, allied health professionals, nurses, or alternative care providers who were not involved in suspicion, first medical consultation, or referral visits.

#### Tests

Parents were asked whether standard screening or diagnosis tests were performed upstream of the initial orthopaedics visit. All parents were asked whether radiographic examination of their child’s back and Adams Forward Bending Test had been performed.

#### Referral visit

The last important milestone in the pathway was the circumstances of referral to orthopaedics. When the originator of the referral request was a health professional, this variable was classified as ‘same as suspicion’, ‘same as first consultation’, ‘regular source of care’, ‘other specialist’, or ‘other medical doctor/professional’ (not involved in previous pathway steps). On the other hand, we counted a minority of cases of lay referrals or ‘self-referrals’, namely, families who obtained a consultation in orthopaedics by their own means, without a prior visit to a primary healthcare provider.

For each of these steps, parents were asked for the names and office locations of the professionals they had seen. We subsequently verified the accuracy of respondents’ categorisation by confirming the specialty of each named professional in the records of Quebec’s medical and professional associations (names were subsequently discarded to preserve confidentiality). The parents also provided approximate dates of visits, using a calendar to determine the sequence of events.

Other variables in the conceptual framework are described below.

#### Predisposing variables

a) scoliosis risk factors: gender, age, family history of scoliosis, location of main scoliosis curve; b) general health: taking regular medication, co-morbidities; c) health-related attitude, behaviour, and knowledge: level of physical activity, general knowledge about scoliosis; d) family socio-economic status: mother’s country of origin (immigration status), mother’s education level; e) family structure and dynamics.

#### Enabling variables

a) family dependent: family annual gross income and region of residence at time of visit in orthopaedics; b) system dependent: density of healthcare resources in the administrative region of the child’s residence (number of paediatricians and number of general practitioners per 1000 inhabitants) and size of orthopaedic clinics.

#### Perceived morbidity (need variables)

a) seriousness; b) urgency: patients and parents were independently asked to indicate on four-point scales their perception, at the time of scoliosis suspicion or detection, of the seriousness of the back problem and of the urgency of consulting a physician. Agreement between the child’s and the parent’s perceived levels of seriousness and urgency was also described in four categories.

### Data analysis

To address the first objective, characterisation of the healthcare pathway, we defined a taxonomy [47]. To achieve this, we first investigated relationships between the categories of the five nominal variables representing healthcare pathway steps, using multiple correspondence analysis (MCA), a form of factorial analysis. One hundred and sixty-six different combinations of pathway steps were empirically observed in our study sample. Use of MCA resulted in data simplification and noise reduction, by searching for common attributes of the observed data, without significant loss of information. Using this technique, a lower-dimension factor subspace was identified, accounting for most of the variance in the data. Participants were thereafter represented by their coordinates on the factorial axes. The decision on the number of factorial axes to retain for subsequent analyses was based on the elbow criterion applied to the eigenvalue curve [48–50], and on the cumulative inertia (adjusted eigenvalues with the Benzécri correction for MCA) [51].

We then used ascending hierarchical classification (AHC) to establish a taxonomy of the pathways, by grouping the participants [52] based on the common characteristics of their pathway steps (similar positioning on the factorial axes). This group partitioning technique involves minimising intra-class variance and maximising inter-class variance. The classification algorithm consisted in grouping together the two individuals closest in space, then iteratively merging the two closest groups of individuals (according to the Ward distance) until all the data were merged into a single class. Each level of the resulting tree was a possible segmentation of the data. The decision on the number of classes to be retained was based on computed change in inter-class inertia (a measure of variability that we wished to maximise) going from n to n-1 class configurations, as well as on the theoretical plausibility, interpretability, and stability of the solution [48]. The final set of classes was considered to represent the taxonomy of pathways and a name was attributed to each class.

In MCA and AHC, supplementary variables (predisposing, enabling, and perceived morbidity factors) from the conceptual framework are not used to compute the factorial axes nor for classification. However, they are useful to the interpretation of results [49]. As such, supplementary variables significantly associated with the classes were used to describe patient profiles within each class.

For the second objective (associations between healthcare pathways and appropriateness of referral), the categories of the newly defined pathway taxonomy were cross-tabulated with the appropriateness of referral status. In addition, adjusted multinomial logistic regression models of these associations were built from operator-specified hierarchical variable blocks involving the previously mentioned predisposing, enabling, and perceived morbidity factors. We presented the most parsimonious model retaining the variables that had a substantial impact on the odds ratios (using a percentage of excess risk of 10 %) [53].

Analyses were carried out with SPAD 7.4 (*Système Pour l’Analyse des Données*, Coheris, France) and IBM SPSS Statistics version 20.

## Results

### Sample characteristics

Of the 930 consecutive eligible children seen in the five participating clinics, 831 completed the study with their accompanying parent, for a participation rate of 89 %. Only 13 eligible families refused to participate. Another 86 eligible children/parents did not complete the questionnaires due to time constraints at the clinic. Therefore, their pathways were not characterized. But since they granted access to their clinical data, we assessed the possibility of selection bias. In fact, they did not differ from the full participants on distribution of available data: appropriateness of referral (main outcome), age, gender, or access to a regular source of care. The study sample (*n* = 831) consisted of 599 girls and 232 boys with a mean age of 13.9 ± 1.9 years. Sixty-three per cent of participants reported having access to a regular source of care, but 31 % declared they had never heard of scoliosis before the initial orthopaedic consultation. The great majority of the children’s mothers had at least a high-school level of education and were born in Canada. Most families resided outside the administrative regions where the orthopaedic clinics were located and in regions where the density of healthcare resources is below average.

There were 517 (62.2 %) children with confirmed AIS, mostly double and thoracolumbar curves with mean Cobb of 26.2 ± 13.3 [11°–91°]. Of these 517 children with AIS, 147 (17.7 % of total sample) were considered late referrals. The remaining 314 children (37.8 % of the total sample) did not meet the diagnosis criteria and were considered to have been inappropriately referred to orthopaedics.

Table 1 shows the distribution of the participants along the five main steps of the healthcare pathway. The suspicion of the presence of a back problem was mostly attributable to laypersons. The first medical consultation for the back problem was mainly (*n* = 417) conducted by the regular source of care (whether at time of suspicion or not). Sixty-nine per cent of patients had undergone x-ray evaluation (with or without bending) before the initial orthopaedic consultation. The great majority of patients had experienced some form of continuity of care leading up to the orthopaedics referral, but 22 % were referred by other doctors, professionals, or specialists, or were self-referred. Three per cent of patients had never had a medical visit before the initial orthopaedic consultation.

**Table 1:** Pathway taxonomy: class composition (frequency, percentage of participants presenting the category within the class)

Variables	Frequency n (%)	Percentage of participants within the given category in the pathway class				
		Pathway 1 Lay/regular source of care interrelation	Pathway 2 Other professionals	Pathway 3 Lay/consultation discontinuity	Pathway 4 Other medical doctor	Pathway 5 Regular source of care continuity
		(*n* = 207)	(*n* = 72)	(*n* = 237)	(*n* = 84)	(*n* = 231)
Suspicion						
Lay	441 (53.5 %)	83.6 % *	72.2 % *	91.1 % *	0	0
Regular source of care	215 (25.9 %)	0	0	0	- -	84.0 % *
Other MD	98 (11.8 %)	0	0	0	72.6 %*	16.0 % *
Other professional	70 (8.4 %)	14.5 % *	25.0 % *	8.9 %	- -	0
First medical consultation						
Same as suspicion	313 (37.7 %)	0	0	0	97.6 % *	100.0 % *
Other MD	262 (31.5 %)	0	33.3 %	100.0 % *	- -	0
Regular source of care	202 (24.3 %)	83.1 % *	41.7 % *	0	0	0
Never	28 (3.4 %)	8.7 % *	13.9 % *	0	0	0
Missing	26 (3.1 %)	8.2 % *	11.1 % *	0	- -	0
Other consultations						
None	608 (73.2 %)	76.3 %	- -	21.9 %	23.8 %	90.9 % *
One	157 (18.9 %)	21.3 %	37.5 % *	- -	- -	- -
More than one	61 (7.3 %)	0	16.7 % *	15.6 % *	- -	- -
Tests						
X-rays	471 (56.7 %)	- -	63.9 %	58.7 %	64.3 %	53.3 %
Bending	183 (22.0 %)	- -	- -	- -	- -	32.5 %*
Both tests	104 (12.5 %)	- -	13.9 %	14.4 %	14.4 %	12.1 %
No	51 (6.1 %)	10.1 %*	6.9 %	6.3 %	7.1 %	- -
Missing	22 (2.6 %)	6.3 %*	0	0	7.1 % *	0
Referral visit						
Same as suspicion	251 (30.2 %)	0	- -	0	- -	99.6 % *
Regular source of care	213 (25.6 %)	83.6 % *	30.6 %	0	- -	0
Same as first consultation	185 (22.3 %)	0	0	78.1 % *	0	0
Other MD or professional	92 (11.1 %)	0	34.7 % *	- -	47.6 % *	0
Self-referral	46 (5.6 %)	16.4 % *	12.5 % *	0	- -	0
Other specialist	41 (5.0 %)	0	9.7 % *	10.6 % *	10.7 % *	0

* : Statistically significant association between the variable category and the class, *p* < 0.05

- - : The given category is not represented in the pathway class

### Healthcare pathway taxonomy

Two factorial axes were retained for the MCA, accounting for 85.1 % of the total inertia in the data [51]. The solution showed that two pathway steps were chiefly responsible for differentiating the participants. The first axis was clearly defined by circumstances of ‘scoliosis suspicion’, from ‘suspicion by the regular source of care’ and ‘other medical doctor’ to ‘lay suspicion’ and ‘other professional’. The ‘referral visit’ step was the main contributor to the second axis, ‘same as first consultation’ and ‘other specialist’ to ‘regular source of care’ and ‘self-referral’. The hierarchical classification algorithm led to a five-class solution; no significant gain in inertia ratio was obtained for less parsimonious solutions. Table 1 presents the pathway class composition and shows percentages of participants presenting a given variable category for each class.

The pathway obtained from Class 1 (termed ‘Lay/regular source of care interrelation’) illustrated the complementary roles of the ‘layperson’ and the ‘regular source of care’ variables. Suspicion was mainly attributable to ‘laypersons’ (or ‘other professionals’), the first medical consultation and the referral visit were mainly conducted with the regular source of care (or never occurred), and ‘no test’ was the most common category for the diagnostic tests. The category ‘somewhat serious’ for the parent’s perception of seriousness is related to this class. The Class 2 pathway (termed ‘Other professionals’) illustrated the effect of relying on ‘other professionals’ for suspicion, referral, and other consultation. Only 72 participants belonged to this class. The Class 3 pathway (termed ‘Lay/consultation discontinuity’) described circumstances where lay suspicion led to a first medical consultation and referral by other medical doctors and consultations with several other professionals, in the absence of a regular source of care. Child’s and parent’s perceptions of the back problem as serious and urgent at the time of suspicion were significantly associated with this class. Also represented in this class were patients in the oldest age category, patients who did not live with both parents or with their mother, patients who did not practice any instructed sports, and patients living outside of the administrative areas where the orthopaedic clinics were located. The Class 4 (termed ‘Other MD’) pathway seemed to account for the particular case of suspicion by a medical doctor not involved as the child’s regular source of care. Many of these children were on medication for other health problems; therefore scoliosis detection may have occurred as an incidental finding in the course of other treatment. This class consisted of only a few patients. Finally, the Class 5 pathway (termed ‘Regular source of care continuity’) represented complete management by the regular source of care. The regular source of care (or other MD in some cases) was responsible for the scoliosis detection and ensured consultation and referral. The most common category for ‘other consultation’ was ‘none’, and ‘bending’ was the most frequent for ‘tests’. Patients younger than the sample mean age who visited the medium-sized clinic were represented in this class.

The taxonomy thus constructed led to a strong association in this study between the pathway classes and the appropriateness of referral status (chi-square = 53.06, *p* < 0.001). As seen in Table 2, pathway 3 (‘Lay/consultation discontinuity’) was clearly associated with an increased likelihood of late referral in comparison to ‘Regular source of care continuity’, chosen as the reference pathway. Lack of continuity also led to increased (although not statistically significant) proportions of late referrals in pathways 2 (‘Other professionals’) and 4 (‘Other MD’). Joint involvement of ‘laypersons’ and the ‘regular source of care’ as represented in pathway 1 was not associated with late referrals when looking at bivariate association. However, after adjusting for negative confounding variables, family structure, and perceived seriousness and urgency, there was a statistically significant association. On the other hand, continuity of involvement of the ‘regular source of care’ in pathway 5 may have been associated with an increased likelihood of inappropriate referral in comparison to pathway 3.

**Table 2:** Associations between the categories of the pathway taxonomy and the appropriateness of referral status

Pathway	Frequency (%)			Odds ratio [95 % CI]	
	Inappropriate	Late	Appropriate	Inappropriate vs. Appropriate	Late vs. Appropriate
1-Lay/regular source of care interrelation (*n* = 207)	93 (44.9 %)	29 (14.0 %)	85 (41.1 %)	1.08 ^a^ [0.71–1.63] 1.25 ^b^ [0.79–1.98]	1.68 ^a^ [0.89–3.19] 2.23 ^b^ [1.13–4.42]
2-Other professionals (*n* = 72)	22 (30.6 %)	16 (22.2 %)	34 (47.2 %)	0.65 ^a^ [0.35–1.22] 0.75 ^b^ [0.38–1.49]	2.16 ^a^ [0.98–4.75] 2.98 ^b^ [1.27–6.96]
3-Lay/consultation discontinuity (*n* = 237)	54 (22.8 %)	67 (28.3 %)	116 (48.9 %)	0.45 ^a^ [0.29–0.68] 0.64 ^b^ [0.39–1.02]	2.62 ^a^ [1.49–4.61] 3.11 ^b^ [1.67–5.77]
4-Other MD (*n* = 84)	36 (42.9 %)	14 (16.7 %)	34 (40.5 %)	1.03 ^a^ [0.58–1.81] 1.09 ^b^ [0.58–2.08]	1.69 ^a^ [0.73–3.91] 1.83 ^b^ [0.73–4.64]
5-Regular source of care continuity (*n* = 231)	109 (47.2 %)	21 (9.1 %)	101 (43.7 %)	1.0	1.0

*CI* Confidence Interval

^a-^ Crude odds ratios

^b-^ Adjusted odds ratios for age, gender, mother’s education, family structure, immigration status, knowledge about scoliosis, likelihood of consulting a physician, sports activity, regular medication, place of residence, hospital size, density of healthcare resources, and child’s perception of seriousness and urgency

## Discussion

Healthcare use in the context of AIS, especially upstream of orthopaedic management, has received very little research attention. The general objective of this study was to investigate how pathways of care for patients with suspected scoliosis could be characterised and related to the appropriateness of referral to specialised orthopaedic clinics in a population without school screening programs or any specific intervention programs for early scoliosis detection.

The taxonomy for the pathways was defined using a combination of multiple correspondence analysis and hierarchical classification analysis. We were able to extract two factorial axes related to the circumstances of two important milestones along the patients’ healthcare journey: scoliosis suspicion and referral to orthopaedics. This suggested the presence of different profiles in our sample, which was confirmed by the classification analysis.

Individuals were grouped into five classes, to which we gave representative labels after careful examination of the grouped variable categories. Continuity of care (pathway 5) may prevent late referrals in comparison to the use of uncoordinated healthcare services and a variety of healthcare providers, such as in pathways 3 and 4. However, direct referral on the basis of clinical suspicion of scoliosis, without proper patient evaluation, may result in over-use of specialised orthopaedic healthcare resources.

The observed determinant role of laypersons and of the regular source of care, along the patients’ healthcare journey, suggests different avenues for public health interventions. First, laypersons (including children themselves) are strongly involved in scoliosis suspicion: in 53 % of cases in the present study and even in 71 % of cases in a Norvegian study [17]. Children’s knowledge of scoliosis played a role in the relationship between the pathways and the appropriateness of referral status, as did perceptions of urgency and seriousness. However, even if lay-perceived morbidity is related to the objective measure of curve severity [54] lay suspicion may not result in appropriate referral. Indeed, lay suspicion in pathways 2 and 5 was associated with an increased likelihood of late referral. Informational support on scoliosis and self-detection initiatives such as leaflets distribution [11] and activities to increase awareness in schools and health education or sports activities may help children and parents detect scoliosis symptoms at an early stage.

With a regular source of care, parents and children were able to seek relational, informational, and management continuity [55], which seemed to have a positive impact on referral status. Having a regular source of care clearly reduced uncoordinated contacts with multiple healthcare providers. However, there is an increased likelihood of inappropriate referrals in these circumstances. Medical practitioners, when encountering uncertainties in dealing with a poorly understood health condition, may rapidly seek expert advice. For example, in a study comparing the diagnoses elaborated by the primary care providers with those of orthopaedists, Reeder et al. [16] concluded that 52.6 % of referrals for suspected scoliosis were considered inappropriate by the specialist. A recent survey [56] revealed that family physicians were not sufficiently aware of symptoms, risk factors for scoliosis progression, and existing treatment, and would welcome having a decision support tool for scoliosis management as well as information on referral guidelines and procedures. According to O’Dunn-Orto et al., musculoskeletal examination is an area of weakness among practising physicians. Increasing teaching time is one aspect of the solution, but also patient educators and interactive small group teaching were identified as preferred strategies for undergrad medical education [57].

As seen in pathway 2, physiotherapists and chiropractors, with their extensive musculoskeletal background, may play a valuable role in scoliosis suspicion and may, for example, provide complementary management of the back problem. However, the fact that they are not allowed to refer their patients directly to a specialist may contribute to the complexity of the healthcare pathway, especially if the patient does not have access to a regular source of care. While Canada has a universal healthcare system, a shortage of resources in the public sector prevents the timely delivery of rehabilitation services. Parents may subscribe to complementary insurance plans to access private services, which may present additional barriers to such care. Collectively defined guidelines could improve interprofessional collaboration and provide much-needed explicit clarification of the role of allied health professionals in scoliosis patient management. Another factor is the involvement of medical doctors other than the regular source of care in scoliosis suspicion, as some patients may be followed by various physicians for other healthcare problems (pathway 4). We believe it is essential that all health professionals who see children and adolescents in their case-mix be aware of scoliosis and have basic information on when it is important to refer these patients to an orthopaedic specialist. Continuing education in musculoskeletal health for health professionals may improve their practice.

From the classification analysis, we found that the bending test for visual detection of trunk asymmetry (without an objective measure) was most often used in pathway 5—a pathway associated with inappropriate referral. It has already been established that this test has good sensitivity but limited specificity [58, 59]. On the other hand, a great proportion of patients (69 %) underwent spinal radiography upstream of the orthopaedic consultation, but the impact of this on the improvement of appropriateness of referral is uncertain. It was observed that patients who had not received prior X-ray examinations were more likely to be inappropriately referred and that X-rays were most frequently done in young adolescents presenting with larger curves, with 80 % of X-rays done in late referrals. In addition, one may raise concerns about the technical quality of the images and the validity of the readings in settings outside of diagnostic facilities. We therefore suggest that valid and reliable screening modalities, such as an inclinometer (the scoliometer) [59–62], should be made available and easy to use for primary care providers. Information on recommended thresholds for referral and referral modalities for specialised care should be more effectively disseminated [63].

### Methodological strengths and limitations

Although a cross-sectional design was used for this study, the temporal sequence of events allowed for logical inferences. Nevertheless, the participants’ perception of morbidity may have changed between the time of suspicion or detection and the referral visit (when it is measured), depending on the nature of their experiences along the healthcare pathway.

The milestones of the healthcare pathway were determined from self-reported information. This type of data, which is usually considered valid, is much more informative than relying on administrative data. While there is potential for recall problems, especially in participants reporting a complex history of healthcare use, we have no reason to suspect the presence of any specific pattern of modification in the responses. Similarly, although social desirability bias could not be ruled out in parental reporting of the initial notification of the scoliosis signs, precautions were taken (by controlling the order of questions, interview approach, etc.) to elicit unbiased collaboration from respondents.

We used multiple correspondence analysis in conjunction with hierarchical classification, an approach to studying interrelationships between categorical variables that is gaining popularity in epidemiology and public health research areas. Here it makes an important conceptual contribution to the field of healthcare utilisation for scoliosis, since it allows a holistic view of the phenomenon, which the study of the individual pathway steps would not have provided. The interpretability and robustness of the solutions, as confirmed by sensitivity analysis, supported our decisions regarding number of factors and chosen classes.

The criteria and cut-off values used to define the outcome categories (appropriate, late and inappropriate referral) were defined from expert opinions based on diagnostic criteria, known risk factors of curve progression and published indications for treatment [20, 21, 28–39]. In absence of guidelines for scoliosis referral, the practice and opinions may vary; therefore, cut-off values of the chosen categories could be discussed. No detailed sensitivity studies were conducted on our regression models to assess this, but a few scenarios were tested without significant impact on the results of such “misclassification” (e.g. classification of mature patients with mild curves in the inappropriate category or moving the cut-off value for the appropriate category to 15 degrees Cobb angle).

The proportions of inappropriate, late, and appropriate referrals in this study were identical to those previously reported for the same geographical area [13]. These findings suggest the possible impacts of having discontinued school scoliosis screening programs and of relying on familial and systemic awareness for scoliosis detection. Indeed, the current case-mix clearly differs from what was observed during the time of school scoliosis screening programs in Quebec [2, 3]. Similarly, Ali-Fazal et al. [15] reported that, at their institution, AIS was most commonly detected by laypersons and that this had increased since 1985 (i.e., after the UK concluded that screening should no longer be a national policy). At the time of their initial visit to an orthopaedic clinic, 70 % of the patients studied had a Cobb angle of more than 40°, as opposed to 28 % in 1985 [14].

The chosen recruitment period was unlikely to have been affected by changes in consultation volume or referral management, particularly as we did not have contact with the referring professionals. Selection bias should be limited since all referred patients were considered for inclusion in the study, the participation rate was high (89 %), non-participants did not differ from full participants on main outcome and on selected available variables. Our participants are believed to be representative of the children and adolescents consulting in paediatric orthopaedics in Quebec since the participating centres reach 70 % of this target population and that we have no reason to believe that the natural history of scoliosis or the referral patterns differ significantly in the rest of the province. Finally, we believe the results are generalizable to contexts where similar (universal) healthcare systems exist. In contexts where accessibility is a significant challenge, the role of laypersons could be even more important and could represent the primary focus for intervention.

## Conclusions

The definition of a taxonomy of five distinct healthcare pathways for children and adolescents with suspected scoliosis offers a conceptual basis to describe the patterns of care utilization upstream of the first orthopaedic consultation, to differentiate healthcare pathways and to study relationships with specialised care utilisation. The method used is original and easily generalizable to other specialised healthcare contexts.

The identification of associations between these healthcare pathways and the appropriateness of referral to specialised orthopaedic clinics, highlighted the circumstances in which over-referral and unmet healthcare needs occurred. The cardinal characteristics that differentiated healthcare pathways and that were significantly related to the appropriateness of referral status, namely the role of laypersons and the involvement of the regular source of care, suggest two main directions for intervention. The first is to increase awareness of signs and symptoms among children, parents, teachers, and professionals involved in adolescent life. Indeed, the role of the lay persons in AIS suspicion was central in a majority of patients. The second is to advocate for access to a regular source of care for all children and adolescents and to improve interactions between the first and second lines of care. The continuity of care is likely to prevent late referrals as opposed to multiple uncoordinated consultations.

The findings of this study contribute significantly to knowledge about the detection, management, and referral circumstances upstream of the consultation in orthopaedics for suspected cases of AIS, and may help in developing strategies for more appropriate referrals in this young population.

Future lines of research would include the further exploration of determinants of the identified pathways. The identification of less favourable pathways shed light on the existence of health care inequalities. Qualitative methods may contribute to better understanding of the barriers to appropriate and timely access at the individual level (attitudes, knowledge, resources, etc.) and at the system level (information, geographical accessibility, volume, etc.) as well as the circumstances leading to the occurrence of less favourable pathways. Research should support the development and evaluation of new interventions to address these inequalities. These new modes of intervention should include support to primary care providers in decision making and promotion of the role of the lay persons.

## Abbreviations

AHC: Ascending hierarchical classification
AIS: Adolescent Idiopathic Scoliosis
BrAIST: Bracing in Adolescent Idiopathic Scoliosis Trial
CI: Confidence interval
MCA: Multiple correspondence analysis
MD: Medical doctor
OR: Odds ratio
SES: Socio-economic status
